# Zinc-alkaline phosphatase at sites of aortic calcification

**DOI:** 10.1007/s10735-024-10207-3

**Published:** 2024-06-08

**Authors:** Santiago Gomez, José Luis Millán

**Affiliations:** 1 Departamento Anatomía Patológica, Facultad de Medicina, Universidad de Cádiz, Plaza Fragela 9, Cádiz, 11003 Spain; 2 Sanford Children’s Health Research Center, Sanford Burnham Prebys Medical Discovery Institute, La Jolla, CA USA

**Keywords:** Zinc, Alkaline phosphatase, Calcification, Aorta, Cartilage

## Abstract

Zinc (Zn) is a normal trace element in mineralizing tissues, but it is unclear whether it is primarily bound to the mineral phase or to organic molecules involved in the mineralization process, or both. Tissue-nonspecific alkaline phosphatase (TNAP) is a Zn metalloenzyme with two Zn ions bound to the M1 and M2 catalytic sites that functions to control the phosphate/pyrophosphate ratio during biomineralization. Here, we studied aortas from Tagln-Cre ^+/−^; Hprt^ALP/Y^ TNAP overexpressor (TNAP-OE) mice that develop severe calcification. Zn histochemistry was performed using the sulfide-silver staining method in combination with a Zn partial extraction procedure to localize mineral-bound (mineral Zn) and TNAP-bound Zn (tenacious Zn), since soluble Zn (loose Zn) is extracted during fixation of the specimens. Two synthetic bone mineral composites with different Zn content, bone ash, and rat epiphyseal growth plate cartilage were used as controls for Zn staining. In order to correlate the distribution of mineral and tenacious Zn with the presence of mineral deposits, the aortas were examined histologically in unstained and stained thin sections using various light microscopy techniques. Our results show that 14 and 30 dpn, TNAP is concentrated in the calcifying matrix and loses Zn as Ca^2+^ progressively displaces Zn^2+^ at the M1 and M2 metal sites. Thus, in addition to its catalytic role TNAP has an additional function at calcifying sites as a Ca-binding protein.

**Graphical Abstract:**

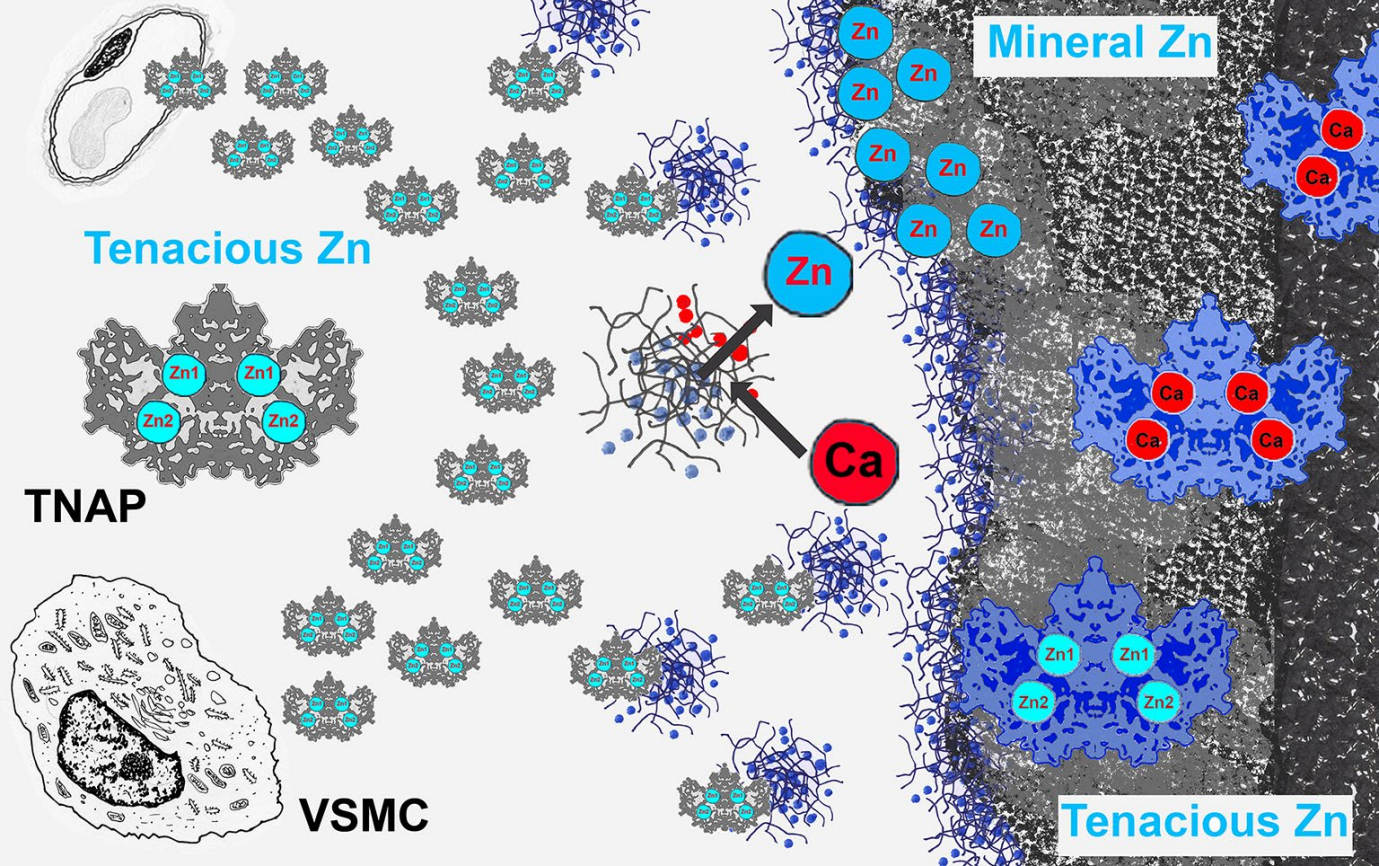

## Introduction

In a hemizygous mouse model overexpressing human tissue-nonspecific alkaline phosphatase (TNAP) in vascular smooth muscle cells (VSMC), Tagln-Cre ^+/−^; Hprt^ALP/Y^, hereafter referred to as male TNAP-OE mice, generalized aortic calcification occurs (Sheen et al. 2015). In this model of arterial calcification, calcification occurs in the arterial media, appears early, progresses rapidly, and is extensive with high TNAP activity observed in plasma (up to 18-fold higher than controls), and enzymatic TNAP activity observed in the arterial wall from post-natal day 7, by histochemistry. At 14 dpn, calcified areas are already present in the aorta of these mice and at 30 dpn the calcifications are severe and the aorta becomes a stiff solidly calcified tube. The mice die prematurely (≈ 44 dpn), as a result of heart failure associated with hypertension and cardiac hypertrophy. Arterial calcification occurs in the absence of systemic changes in calcium, phosphate, pyrophosphate, or renal function. VSMCs of the arterial media manifest chondro-osteogenic differentiation (Sheen et al. 2015).

TNAP is a key enzyme in the control of physiological mineralization of skeletal and dental tissues. Its importance is demonstrated by the impaired mineralization observed in human hypophosphatasia (Millán and Whyte 2016) and in transgenic mouse models that phenocopy the disease (Narisawa et al. 1997; Fedde et al. 1999; Anderson et al. 2005). In mineralizing tissues, TNAP functions both as a pyrophosphatase (cleaving pyrophosphate, a potent inhibitor of mineral growth (Meyer 1984), into two molecules of phosphate) and as a general phosphoesterase (Millán 2006; Wuthier 2011). A calcium-binding function in the cartilage calcification has also been suggested (Vittur et al. 1973; Stagni et al. 1979; De Bernard et al. 1986).

TNAP is a Zn metalloenzyme with two Zn ions anchored to the M1 and M2 catalytic sites. There are two additional metal sites in the molecule, M3 occupied by magnesium (Mg) and a structural M4 occupied by calcium (Ca) (Mornet et al. 2001; Hoylaerts et al. 2015). Kinetic studies in vitro have shown that Ca^2+^ can displace Zn^2+^ and Mg^2+^, occupying these sites to ultimately inactivate the enzyme (Hoylaerts et al. 2015).

Classically, histological studies of TNAP in calcifying tissues localize its distribution by enzymatic staining using glycerol phosphate or azo dye methods. However, as the enzyme is inactivated during the calcification process (Genge et al. 1988), its enzymatic detection by staining in calcifying sites is no longer possible (Bonucci and Gomez 2012). However, TNAP immunohistochemical studies indicate that TNAP protein is still present in recently calcified cartilage (Väänänen 1980; De Bernard et al. 1986) as well as in the calcification front in bone tissue (Gomez et al. 1999; Hoshi et al. 2001). In the present study, we used a histochemical method using Zn partial extraction and sulfide-silver staining to localize Zn-TNAP in the calcified aorta of male TNAP-OE mice (Gomez et al. 1999).

## Materials and methods

### Samples, embedding, and thin-sectioning

Calcified aortas of male TNAP-OE mice. Full details on the generation and genetic analysis of the Tagln-Cre ^+/−^; Hprt^ALP/Y^ mice model have been published previously (Sheen et al. 2015). In short, homozygous Hprt^ALPL/ALPL^ female mice were bred with male mice expressing Cre-recombinase under the control of the VSMC-specific transgelin promoter (Tagln-Cre). All male offspring are hemizygous Hprt^ALPL/Y^; Tagln-Cre^+/−^ (hereafter referred to as male TNAP-OE mice) and expected to display a full overexpression phenotype. Because of the X-linked nature of this transgene and the variability introduced by X-chromosome inactivation, heterozygous Hprt^ALPL/–^; Tagln-Cre^+/–^ female mice display a more variable phenotype and were not used in this study (Sheen et al. 2015).

Thus, five Tagln-Cre ^+/−^; Hprt^ALP/Y^ calcified aortas were used for the current study; two from 14 dpn and three from 30 dpn male TNAP-OE mice. Healthy aortas from 14 dpn and 30 dpn control mice showed no calcifications and no positivity in the histochemical staining for TNAP activity using the azo dye method (Sheen et al. 2015).

Aortas were fixed in 4% paraformaldehyde and embedded undecalcified in poly-methyl methacrylate (PMMA). Thin ground sections (10–50 μm thick) were prepared from each aorta in 6–8 planes using a previously described grinding and polishing technique (Arzac et al. 2018). This method has the advantage of producing artifact-free sections, leaving mineral deposits intact, but the disadvantage of not being able to obtain serial sections. In the best case, two correlative sections of the same PMMA block are at least 500 μm apart.

Zn minerals. Two synthetic bone mineral composites with different Zn content (SBM) #80 and #57 (kindly provided by Raquel Legeros, New York College of Dentistry) and bone ash (Standard Reference Material 1400, National Institute of Standards & Technology, USA) were used as controls for Zn staining. Preliminary analysis by inductively coupled plasma optical emission spectrometry (ICP-OES) determined Zn content for #80 Zn-SBM = 55,150 µg/g and for #57 Zn-SBM = 17 µg/g. Bone ash has a certified Zn content of 181 ± 3 µg/g. SBM and bone ash powders were directly embedded in PMMA and processed in the same manner as aortas.

Zn-TNAP in rat epiphyseal growth plate cartilage. Correlation studies for Zn and TNAP histochemistry were used as a control for Zn-TNAP localization in epiphyseal growth plate cartilage from rapidly growing rats. Growth plate cartilage is an ideal tissue for Zn-TNAP localization because a longitudinal section shows the typical zonal distribution (resting, proliferative, hypertrophic, and calcifying zones) with and without TNAP activity in chondrocytes and extracellular matrix over a distance of a few hundred microns (Bonucci and Gomez 2012). For Zn histochemistry, the right tibiae from nine 20 dpn Wistar rats were fixed in 10% neutral formaldehyde and embedded in glycol methacrylate (GM) without decalcification. For TNAP histochemistry, left tibiae were fixed in 10% neutral formaldehyde for two hours at 4 °C and then embedded without decalcification under vacuum with reduced catalyst for GM at 4 °C as described (Gomez and Boyde 1994). GM blocks were serially sectioned (1.5–2.5 μm thick) using a heavy-duty microtome HM350 (Microm, Walldorf, Germany) equipped with glass knives with a 1-cm cutting edge.

### Zn histochemistry

Zn histochemistry was performed using the sulfide-silver staining method in combination with a Zn partial extraction procedure to localize Zn-TNAP (Gomez et al. 1999). The sulfide-silver staining method (Timm’s method) is a highly sensitive photographic development procedure. It produces fine-grained metallic silver deposits but requires very long development times. It consists of two steps: (1) sulfide exposure of tissues to form a latent image consisting of Zn-S germs (“active centers”); (2) physical development of the latent image by deposition of reduced silver on the Zn-S germs (Gomez et al. 1999; Danscher et al. 2004).

Zn partial extraction was performed prior to sulfide exposure of the samples in two ways. Soluble Zn (labile, loose Zn) was extracted by not fixing the aortas with sulfide (no-sulfide fixation). Additional Zn partial extraction was performed by decalcifying the sections with 10% neutral ethylenediaminetetraacetic acid (EDTA), so that Zn bound to mineral phase (mineral Zn) was also extracted when mineral was removed. After these extractions, the remaining Zn was considered to be Zn with a higher binding strength (firm, tenacious Zn) belonging to metalloenzymes found in the organic matrix.

Sulfide exposure was performed directly on sections prepared from SBM, bone ash, rat epiphyseal cartilage, and calcified aorta samples. The sections were stained according to two procedures as reported (Gomez et al. 1999). In the first procedure (#1 S-Ag procedure), undecalcified sections were treated with a 10% sodium sulfide solution and then physically developed. SBM and bone ash were treated with sulfide in series for 10 to 60 min to determine the optimal exposure time according to their Zn concentration. Calcified aortas were exposed to sulfide for 30 min. In the second (#2 E-S-Ag procedure), undecalcified sections of rat epiphyseal cartilage and mouse aorta were first decalcified with 10% EDTA for 30 min to remove mineral (and additional Zn partial extraction), then exposed to sodium sulfide for 30 min and physically developed in the same manner.

The developer solution was a silver nitrate-hydroquinone reducing solution (pH 4) prepared by mixing 30% gum arabic (60 mL), 5.67% hydroquinone (30 mL), 2 mol/L citrate buffer (10 mL), and 1% silver nitrate (5 mL). Physical development was performed in the dark for 40–120 min at a temperature of 20–23 °C. Developer was changed every 20 min and staining was stopped in running water.

### Histology

To correlate Zn distribution with the presence of mineral deposits in the calcified aortas, the aortas were also examined by conventional histological methods. Thick (50 μm) sections were surface-stained (Schenk et al. 1984; Bromage et al. 2018) either with alizarin red (5 min) or von Kossa (1% silver nitrate, 30 min under black light) for calcium phosphate mineral; or they were acid-etched, 5 min with 1% periodic acid, or decalcified with 10% EDTA, 30 min, then surface-stained with toluidine blue for organic matrix components (proteoglycans, acidic proteins in general). Some unstained thin sections (10 μm) were also prepared and examined microscopically unstained or after staining.

The thin (1.5–2.5 μm) undecalcified sections from GM blocks of rat epiphyseal cartilage processed at 4 °C were stained using a simultaneous azo dye coupling method with naphthol AS phosphate as substrate and Fast blue BB to detect the sites of enzymatic activity of TNAP (Gomez and Boyde 1994), or they were stained with von Kossa for calcium phosphate mineral. These sections were used to correlate the sites of enzyme activity and mineral deposition with the distribution of tenacious Zn in different zones of the growth plate.

### Light microscopy

Surface-stained thick sections of SBM, bone ash, calcified aorta and stained thin sections of rat cartilage were examined with an Optiphot-2 EFD-3 microscope (Nikon, Minato, Japan) equipped for bright field and epifluorescence microscopy. Unstained and stained thin sections of calcified aorta were examined with another Optiphot-2 microscope equipped for cross polarization and positive phase contrast microscopy. In the present study, because the staining layer in the surface-stained sections was a few micrometers thick (≤ 5 μm), these sections, and microtomized thin sections could be observed with a high-magnification, high-resolution Nikon Plan Apo objective (100/1.4) using oil (nd = 1.515). A DXM1200F (Nikon) CCD camera was used for imaging.

## Results

### Histology of TNAP-OE calcified aortas

Microscopic examination of the aorta sections confirmed the presence of calcifications in the arterial media of 14 dpn and 30 dpn male TNAP-OE mice. These calcifications appeared as plaques, small stones, and minute deposits. In more advanced cases, stones protruded into the intima, and minute deposits began to appear in the adventitia. The arterial media was also thickened by the deposition of new non-calcified matrix. Depending on the level and size of aorta studied, three to five elastic laminae in the media were detected.

**Fig. 1:**
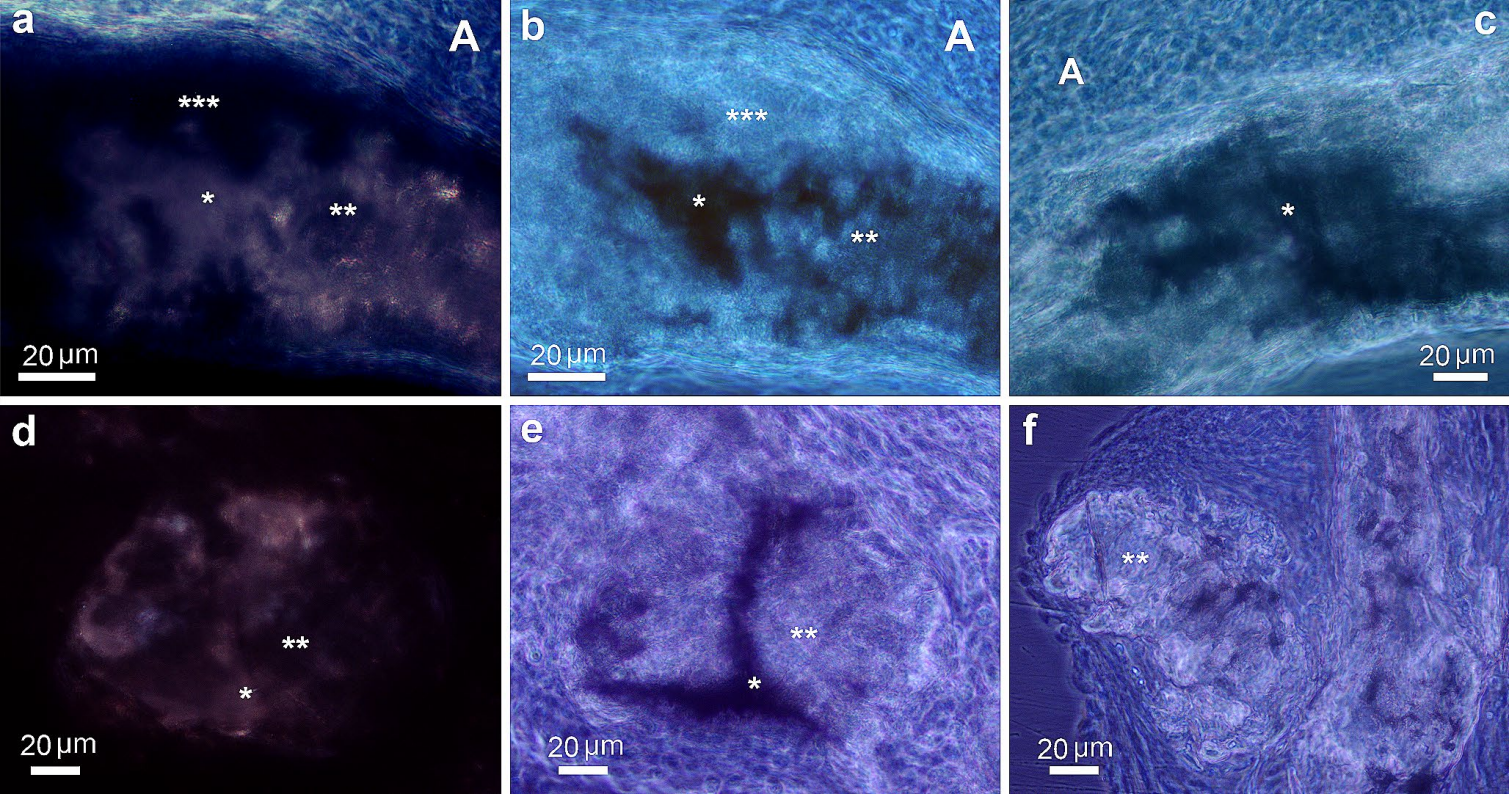
Optical properties of mineral and matrix components of TNAP-OE calcified aortas. (**a**-**f**) Unstained thin sections. (**a**) 14 dpn aorta. Areas of recent mineral deposition (calcifying sites) with brownish areas (*) and dark areas (**). (***) indicates matrix of non-mineralized arterial media and (**A**) adventitia, which appear dark and bright, respectively. Cross polarization microscopy. (**b**) 14 dpn aorta. Same FOV as (**a**) seen by phase contrast microscopy. (*) indicates the darker component (concentration of non-collagenous proteins) and (**) the brighter component (fibrillar collagen). (***) Non-mineralized arterial media and (**A**) adventitia are also brighter than the surrounding gray media. (**c**) 14 dpn aorta. A calcifying area in which the darker component (*) predominates. Phase contrast microscopy. (**d**) 30 dpn aorta. Area of more advanced mineral (calcified) with a higher proportion of black area (**) than brownish (*). Cross polarization microscopy. (**e**) 30 dpn aorta. Same FOV as (**d**) seen with phase contrast microscopy with more bright (**) than dark (*) optical component. (**f**) 30 dpn aorta. A calcified area with a predominance of the brighter component (** fibrillar collagen). Phase contrast microscopy

In unstained thin sections, cross-polarization revealed that early mineral deposits (calcifying) in 14 dpn aortas appeared as a brownish cloud (* in Fig. 1a) with some black areas (** in Fig. 1a). In 30 dpn aortas, more advanced mineral deposits (calcified) showed increasing black zones (** in Fig. 1d) until they were almost completely black.

The same fields of view (FOV) observed with positive phase contrast microscopy revealed the presence of two types of optical components in the mineral deposits. One had a higher refractive index and appeared darker than the surrounding media (* in Fig. 1b-c, e), mineral deposited in a matrix rich in non-collagenous proteins, whereas the other appeared brighter than the gray background, mineral deposited in a matrix rich in collagen fibrils (** in Fig. 1b, e-f). The darker component was abundant in early mineral deposits, still calcifying, found mainly in 14 dpn aortas (* in Fig. 1c), whereas the brighter component was predominant in more calcified deposits in 30 dpn aortas (** in Fig. 1f). These changes observed in the mineralized zones by polarization and phase contrast microscopy were interpreted as the interaction of three components with unequal optical properties. Mineral, a crystalline structure, has intrinsic birefringence, collagen fibers have form birefringence, while non-collagenous proteins are optically opaque.

Under cross polarization, non-mineralized media appeared dark (***in Fig. 1a), while the adventitia, which is mainly composed of collagen fibers, appeared bright (A in Fig. 1a). Phase contrast microscopy of non-mineralized media showed a brighter fibrillar component (*** in Fig. 1b). The aortic adventitia was also a brighter component but showed thicker collagen fibers (A in Fig. 1b-c). Although both the adventitia and the non-mineralized media matrix consisted mainly of a collagenous component, cross-polarization microscopy determined different retardation values in the two cases. In the adventitia, the collagen fibers were thicker and oriented in the plane of section, so they appeared bright. In contrast, the fibrillar component of the media was thinner and disorganized and appeared dark (similar to woven bone). This difference in collagen fiber arrangement and thickness was confirmed by examining the same FOV with 546 nm monochromatic polarization using a 1/4 lambda retarder (data not shown).

**Fig. 2:**
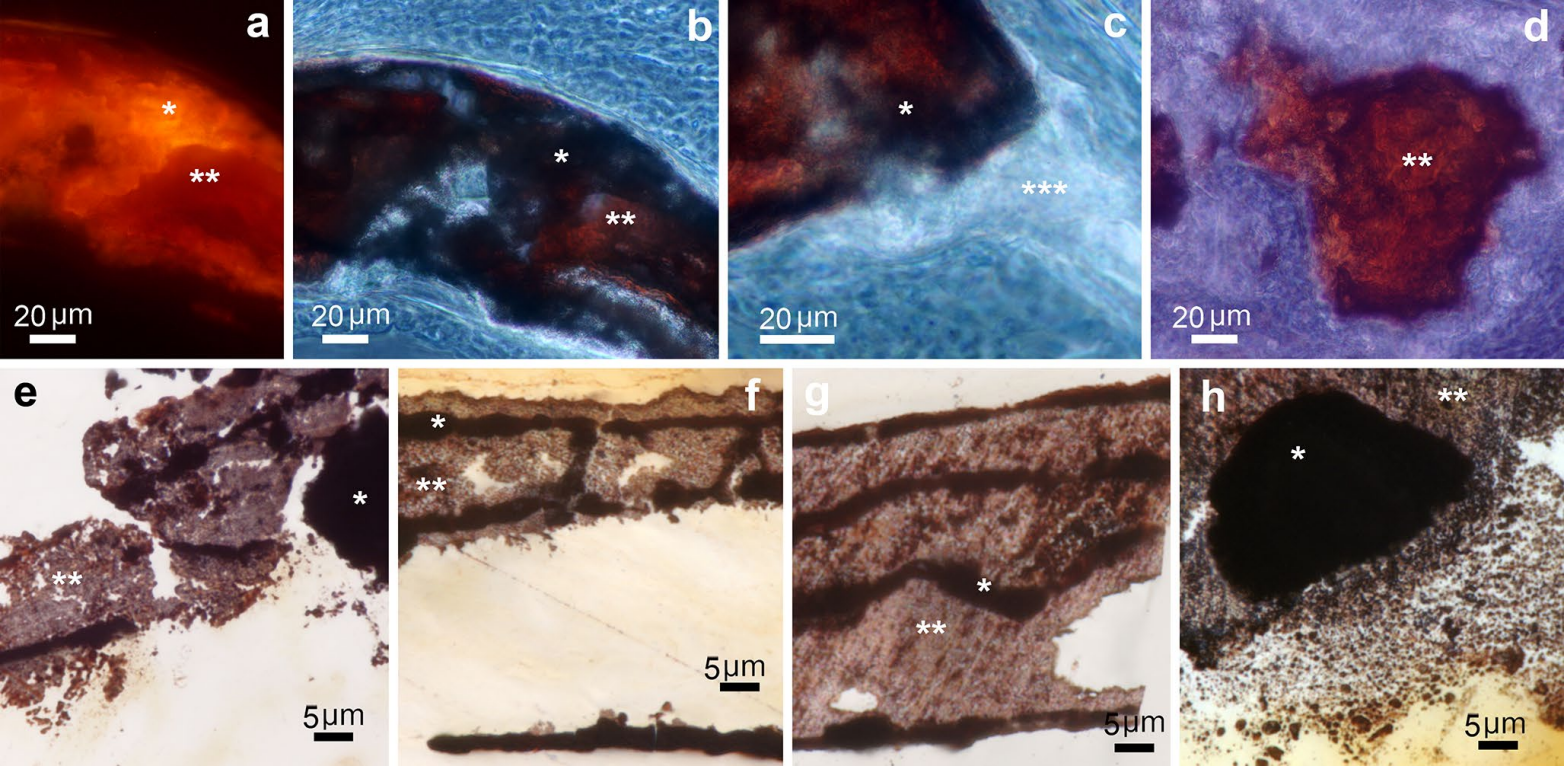
Mineral staining of TNAP-OE calcified aortas. (**a**-**d**) Alizarin red; (**e**-**h**) von Kossa. (**a**) 14 dpn aorta. The calcifying area is unevenly stained with orange (*) and red (**) areas. (**b**) 14 dpn aorta. Same FOV as (**a**) seen with phase contrast microscopy, where (*) indicates the orange area with the same dark component (*) detected in the unstained sections; (**) indicates the area of red staining. (**c**) 14 dpn aorta. Detail of the periphery of a calcifying area (*) showing the dark component. (***) indicates the bright component (fibrillar collagen) in non-mineralized arterial media. (d) 30 dpn aorta. Detail of a calcified area showing that the red staining area contains fibrillar collagen (**). (**e**) 14 dpn aorta. An incompletely calcified sector of the aortic wall with black (*) and brown (**) silver deposits. (**f**) 30 dpn aorta. Detail of an incompletely calcified of the aortic wall aorta showing initial mineral deposits (arrow) on a calcified elastic lamina (*). (**g**) 30 dpn aorta. Detail of a fully calcified sector of the aortic wall (*) showing calcified elastic laminas stained black and (**) brown mineral deposits between them. (**h**) 30 dpn aorta. Detail of an area showing a small stone (*) and minute mineral deposits (**). (**a**) Epifluorescence microscopy using a B-2 A filter cube (Nikon); (**b**-**d**) phase contrast microscopy; (**e**-**h**) bright field microscopy

In calcified aortas, calcium phosphate mineral deposits were stained with alizarin red (Fig. 2a-d) and von Kossa stain (Fig. 2e-h), although unevenly. Alizarin red stained mineral deposits orange-red (Fig. 2a). In thin sections stained with alizarin red, the same FOV observed with phase contrast microscopy showed that the orange zones in calcifying deposits had the same darker optical component as observed in unstained thin sections (* in Fig. 2b-c). In contrast, the fibrillar collagen areas in calcified deposits were stained red (** in Fig. 2d). The fibrillar collagen matrix in the non-calcified media and the collagen fibers in the adventitia were not stained (*** in Fig. 2c).

Von Kossa also stained mineral deposits unevenly with black to brown staining (Fig. 2e-h). Black silver deposition was more common in the 14 dpn aorta (Fig. 2e). Brown zones were found in more advanced calcified deposits and were present in the mineralized plaques in 14 dpn and 30 dpn aortas, occupying the spaces between elastic laminae (Fig. 2e-g). Elastic laminae were completely calcified within the plaques or incompletely calcified outside the plaques (* in Fig. 2e-g). Some early mineral deposits were observed on calcified elastic lamina (Fig. 2f). Small stones and minute deposits were also stained black or brown (* and ** in Fig. 2h).

**Fig. 3:**
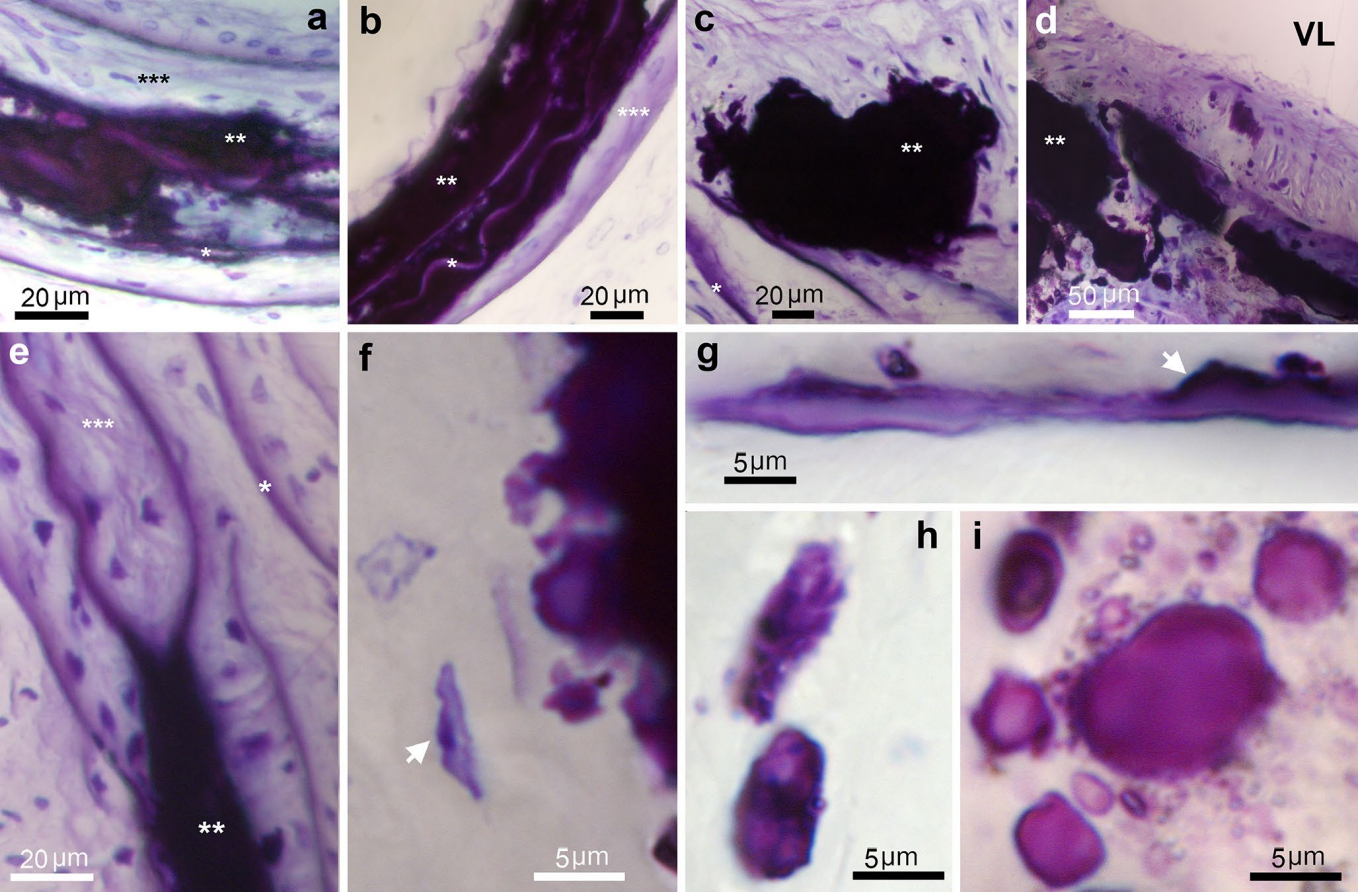
Matrix staining of TNAP-OE calcified aortas. (**a**-**f**) Periodic acid & toluidine blue; (**g**-**i**) EDTA & toluidine blue. (**a**) 14 dpn aorta. Early calcifying areas stained deep blue (**). (*) indicates elastic laminae and (***) non-mineralized media. (**b**) 14 dpn aorta. Calcifying plaque intensely stained blue which is almost completely occupying the arterial media (**). Note that the enclosed elastic laminae (*) are lighter in color. (***) indicates non-mineralized media. (**c**) 30 dpn aorta. Small stone stained deep blue (**). (*) indicates elastic laminae. (**d**) 30 dpn aorta. Panoramic view of the aortic wall showing advanced calcification with deeply stained plaques (**). (**e**) 30 dpn aorta. Detail of a calcified area (**) containing five elastic laminae (*) bordering a non-mineralized area of the arterial media (***). (**f**) 30 dpn aorta. Detail of the periphery of a stone with surrounding VSMCs. VSMC shows stained dots (arrow). (**g**) 30 dpn aorta. Detail of deep blue staining of early deposits (arrow) formed on an elastic lamina. (**h**) 30 dpn aorta. Early mineralization foci showing intense stained dots inside. (**i**) 30 dpn aorta. Calcospherulites with less intense staining inside because they are more calcified. Note that they are bordered by a deep blue rim. (**a**-**i**) Bright field microscopy

After oxidation and decalcification by periodic acid, the organic matrix of the mineral deposits stained deep blue with toluidine fairly homogeneously (** in Fig. 3a-f). VSMCs in the vicinity of the small stones showed equally stained dots (arrow in Fig. 3f). Staining with toluidine blue after decalcification with EDTA gave similar results, although the areas of more advanced calcification stained lighter (Fig. 3g-i). Initial mineral deposits formed on elastic lamina stained deep blue (Fig. 3g), and in extracellular early mineralization foci (about 10 μm in size) the staining was somewhat uneven, with deep blue dots within the minute foci (Fig. 3h). Staining was also less intense in the center of more calcified calcospherulites (Fig. 3i).

### Zn histochemistry of Zn minerals

After physical development, the positivity of the staining for Zn was demonstrated by the fine-grained metallic silver deposition, which showed a black color. Areas with less silver deposition had a paler, gray or brown color, which was interpreted as areas with lower initial ZnS germ content. Zones with no silver deposition indicated no detectable Zn.

**Fig. 4:**
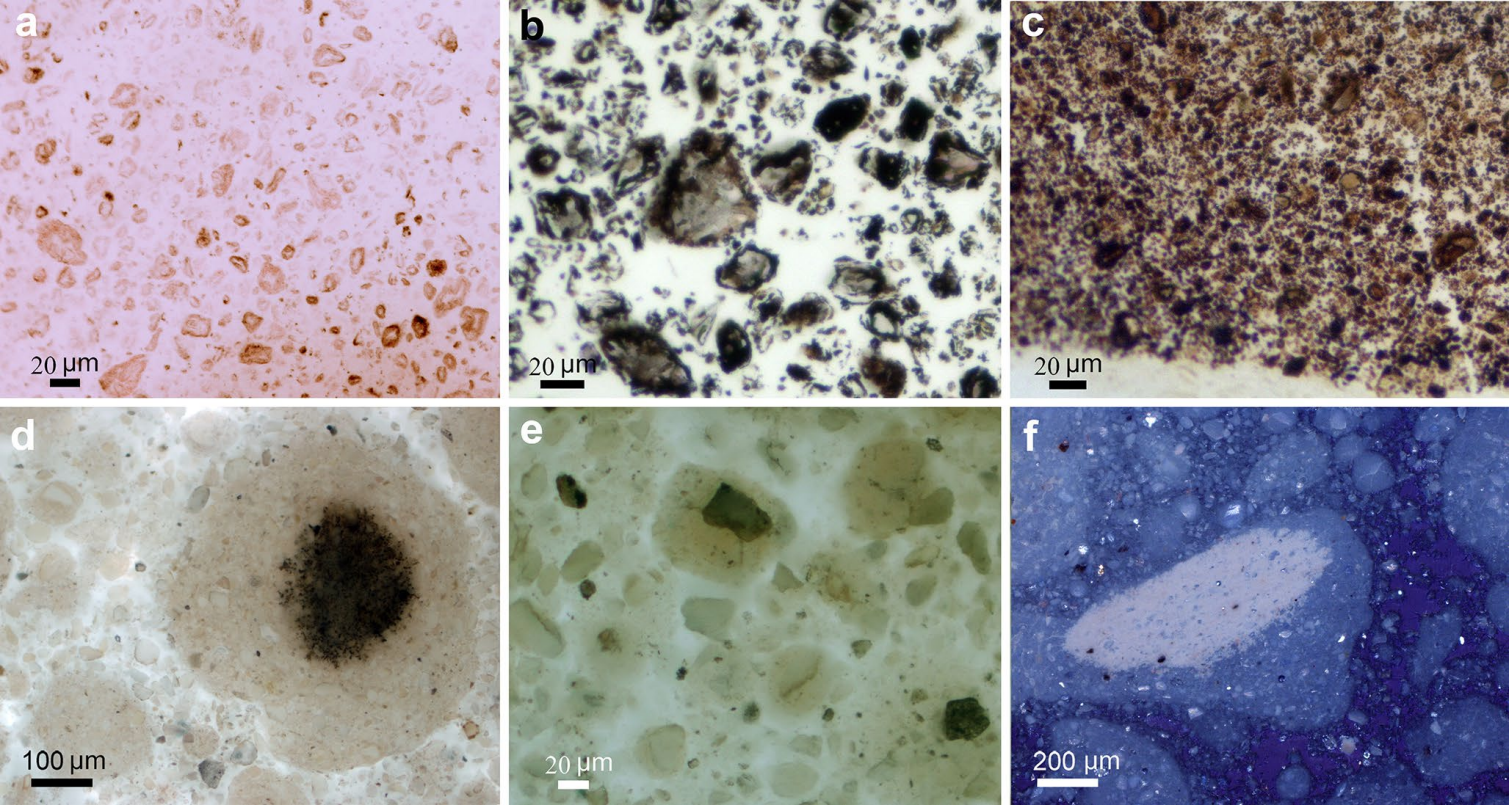
Zn histochemistry of SBM and bone ash. #1 sulfide silver procedure. (**a**) Light brown staining of #57 SBM after sulfide exposure for 10 min and physical development for 40 min. (**b**) Black staining of #80 SBM after sulfide exposure for 10 min and physical development for 40 min. (**c**) More intense staining of #57 SBM after sulfide exposure for 60 min and physical development for 60 min. (**d**-**e**) Focal Zn staining of bone ash after sulfide exposure for 60 min and physical development for 60 min. (**f**) Bone ash viewed with polarization shows gray and white interference colors because it is composed of different mineral phases. Note that the white areas correspond to zones of higher Zn concentration in Fig. 4d. (**a**-**e**) Bright field microscopy; (**f**) Cross polarization microscopy

Zn histochemistry using the #1 S-Ag procedure showed uniform silver staining in both SBM, but a focal distribution of silver deposits in bone ash (Fig. 4a-e). All of this stained Zn was considered to be mineral-bound Zn (mineral Zn). However, both SBM #57 (Zn content = 17 µg/g) (Fig. 4a, c) and bone ash (Zn content = 181 µg/g) (Fig. 4d-e) required longer sulfide exposure and development time than SBM #80 (Zn content = 55,150 µg/g) (Fig. 4b), indicating that the initial Zn concentration, and thus the number of ZnS germs formed, was a determining factor in the deposition of metallic silver during physical development. Heterogeneity of bone ash was also observed by polarization microscopy (Fig. 4f) and may be due to different mineral phases formed during heating of the bone at 1100 °C.

### Zn and TNAP histochemistry of rat epiphyseal growth plate

**Fig. 5:**
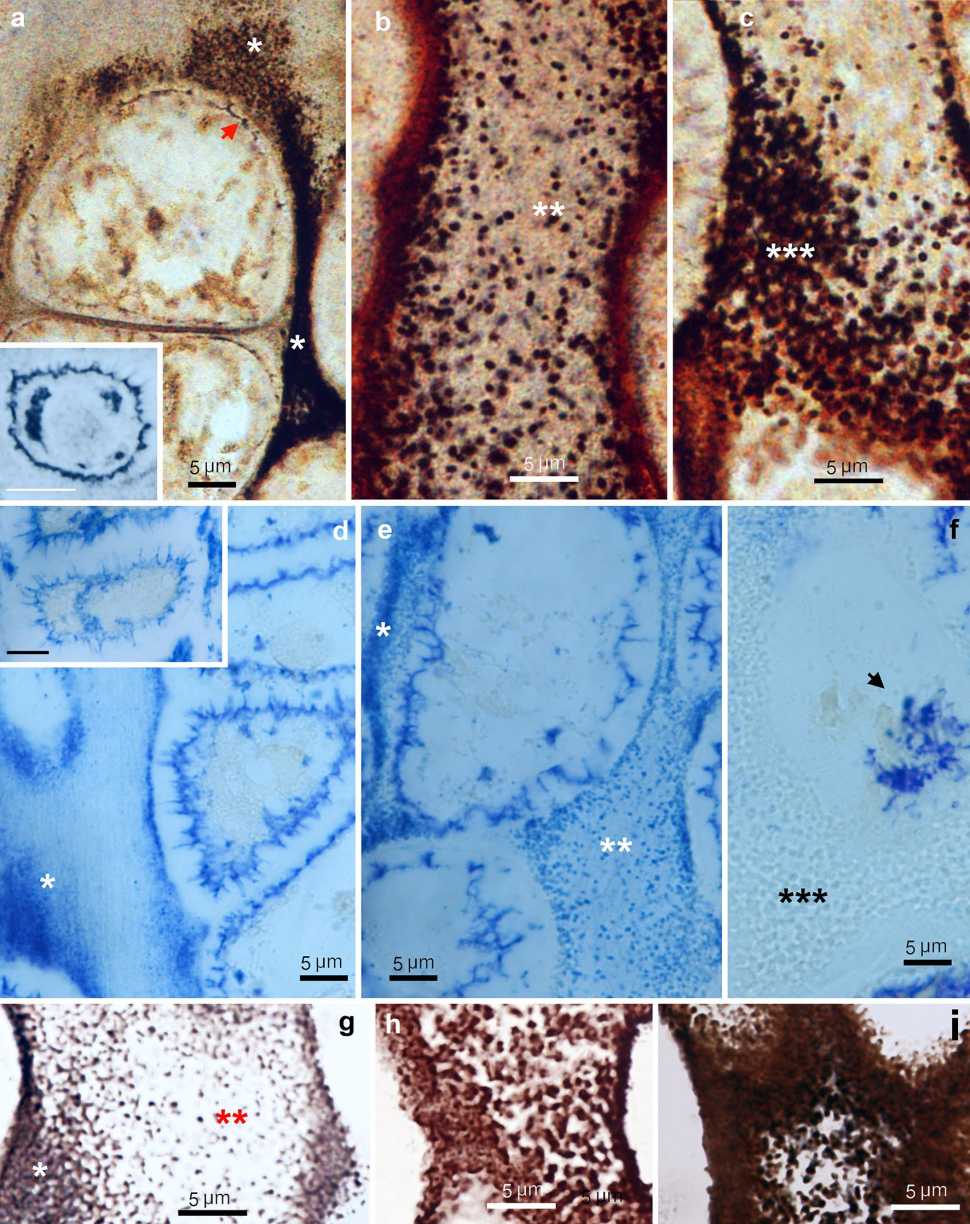
Rat epiphyseal growth plate. (**a**-**c**) Zn histochemistry, #2 EDTA sulfide silver procedure. (**d**-**f**) TNAP histochemistry (in blue). (**g**-**i**) Von Kossa stain. (**a**) Hypertrophic zone. (*) indicates territorial matrix. Arrow indicates plasma membrane staining. 40 min of physical development. Inset, a chondrocyte located in the upper hypertrophic zone shows plasma membrane and intracellular staining after 90 min of physical development, scale bar 5 μm. (**b**) Calcifying zone. (**) indicates the presence of numerous dots corresponding to initial calcification nodules in the interterritorial matrix. (**c**) Calcifying zone. (***) indicates confluent calcification nodules. (**d**) Hypertrophic zone. (*) indicates positivity in the territorial matrix. Inset, a chondrocyte located in the upper hypertrophic zone shows positivity in the plasma membrane and cytoplasmic deposits, scale bar 5 μm. (**e**) Calcifying zone. (**) indicates the presence of numerous dots corresponding to initial calcification nodules in the interterritorial matrix. (**f**) Lower border of the calcifying zone with confluence of calcification nodules (***). Note that the enzymatic activity of TNAP disappears in the calcified deposits. Arrow indicates an apoptotic chondrocyte with shrunken cytoplasm. (**g**) Calcifying zone. (*) indicates early calcification in the territorial matrix. (**) indicates numerous small dots in the interterritorial matrix corresponding to initial calcification nodules. (**h**) Lower border of calcifying zone, calcification nodules are larger and confluent. (**i**) Lower border of the calcifying zone with completely calcified septa. (**a**-**i**) Bright field microscopy

Decalcified cartilage sections, #2 E-S-Ag procedure (Fig. 5a-c). In the growth plate, after 40 min of development, silver deposits were located in the territorial matrix of the lower hypertrophic zone as a cloud of black grains or as a black septum, depending on the incidence of the section plane (* in Fig. 5a). The plasma membrane of the lower hypertrophic chondrocytes was focally stained (arrow in Fig. 5a). With longer development times (90 min), the plasma membrane was stained completely together with some cytoplasmic deposits in both upper (inset in Fig. 5a) and lower hypertrophic chondrocytes. In the calcifying zone, silver deposits appeared as numerous dots (0.4–0.6 µm in size) in the interterritorial matrix of the longitudinal septa (** in Fig. 5b). At the lower border of the calcifying zone, the silver dots were larger and more confluent (*** in Fig. 5c).

TNAP histochemistry (Fig. 5d-f). In the growth plate, TNAP was expressed early in the plasma membrane of the first chondrocytes emerging from the proliferative zone. In the hypertrophic zone, TNAP was already present in the territorial extracellular matrix (* in Fig. 5d) and in the plasma membrane and in cytoplasmic deposits of chondrocytes (inset in Fig. 5d). In the calcifying zone, TNAP was localized as numerous scattered dots (0.4–0.6 μm in size) in the interterritorial matrix of the longitudinal septa (** in Fig. 5e). At the lower border of calcifying zone, where apoptotic chondrocytes were found (arrow in Fig. 5f), TNAP was negative in the confluent calcification nodules and calcified septum (*** in Fig. 5f). Complementary von Kossa staining of serial sections (Fig. 5g-i) showed that the initial calcification nodules, optically visible at high magnification, were located in the calcifying zone, around chondrocytes in the territorial matrix (* in Fig. 5g) and as small dots, 0.3–0.6 μm in size, in the interterritorial matrix of the longitudinal septum (** in Fig. 5g). At the lower border of calcifying zone, calcification nodules were larger (0.7–2 μm in size) and confluent to form a calcified septum (Fig. 5h, i).

### Zn histochemistry of TNAP-OE calcified aortas

**Fig. 6:**
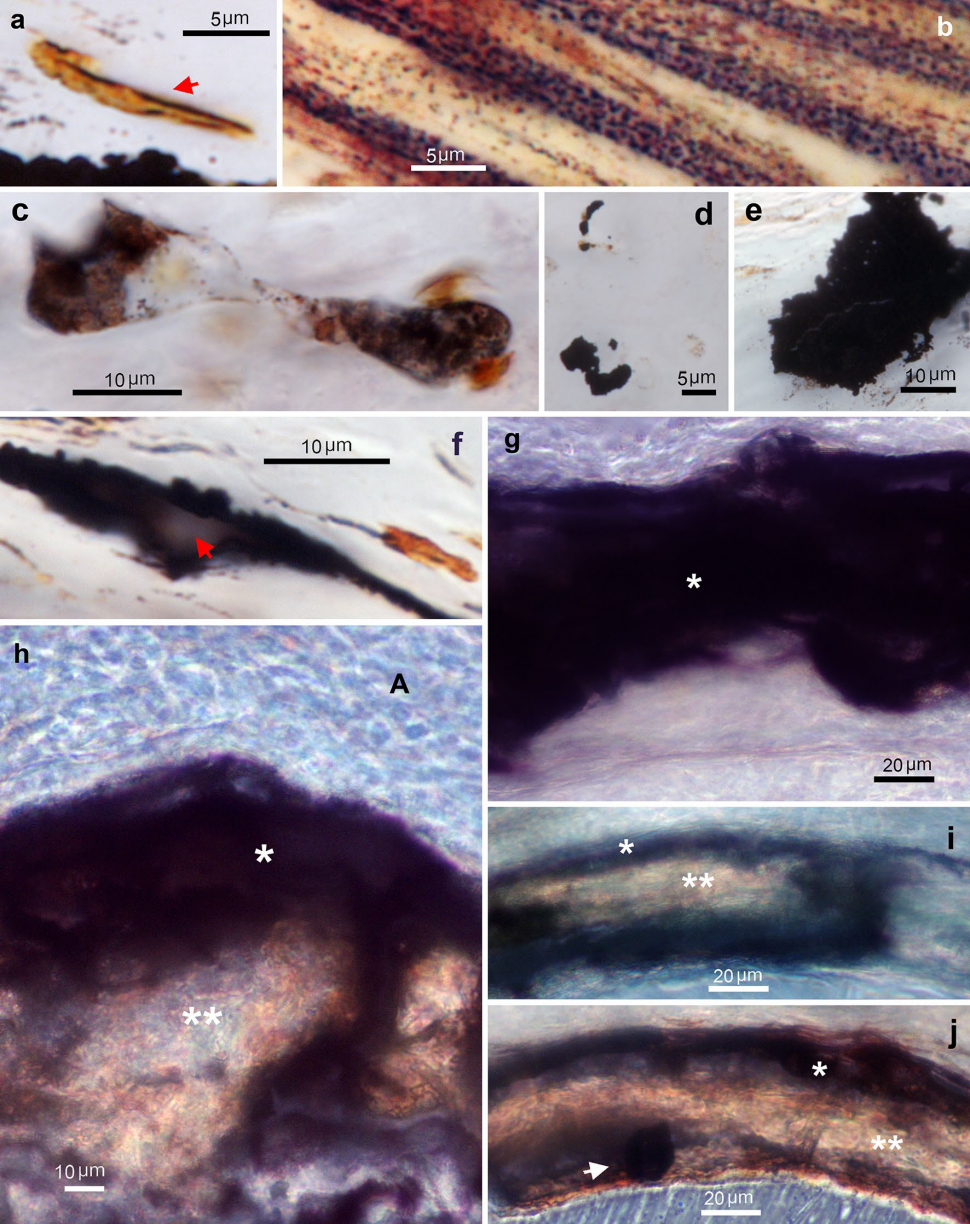
Zn histochemistry of TNAP-OE calcified aortas. #1 sulfide silver procedure. (**a**) 30 dpn aorta. VSMC with black linear deposits on its outer membrane (arrow). (**b**) 14 dpn aorta. Elastic laminae with numerous stained dots. (**c**) 30 dpn aorta. Two early mineralization foci with uneven brown and black staining. (**d**) 30 dpn aorta. Early mineralization foci stained completely black. (**e**) 30 dpn aorta. Small stone completely stained black. (**f**) 30 dpn aorta. Early mineral deposit on elastic lamina, both stained black. Note that the center of the mineral deposit is lighter (arrow). (**g**) 14 dpn aorta. Fully stained calcifying areas (*) occupying almost the entire thickness of the arterial media. (**h**) 14 dpn aorta. Another area of the same aorta section as (**g**) with more advanced calcification. The calcifying areas (*), of irregular distribution, surround a calcified center (**) with little staining. (**i**) 30 dpn aorta. Calcified plaque (**) surrounded by calcifying areas with black staining (*). On the right is an area of the arterial media that is not calcified and not stained. (**j**) 30 dpn aorta. Calcified plaque (**) with brownish staining. The calcifying areas, in black, are mainly in the outer peripheral part. The arrow points to a small stone, stained black, trapped within the plaque. (**a**-**f**) Bright field microscopy. (**g**-**j**) Phase contrast microscopy

Undecalcified aorta sections, #1 S-Ag method (Fig. 6). In non-mineralized arterial media, VCMSs were stained at the plasma membrane (arrow in Fig. 6a). Elastic laminae and some fibrillar collagen were completely or incompletely stained (Fig. 6b). Early mineralization foci about 10 μm in size (as described in toluidine blue staining section) were unevenly stained, dark brown with black dots inside (Fig. 6c), while others were black (Fig. 6d). Small stones scattered throughout the matrix stained black (Fig. 6e). Small mineral deposits formed on the elastic lamina were also stained black but had a gray center (arrow in Fig. 6f). Calcifying areas in 14 dpn and 30 dpn aortas were stained black (* in Fig. 6g-j), whereas more advanced calcified areas were either unstained or light brown (** in Fig. 6h-j). The distribution of calcifying areas in the arterial media was irregular. They were found patchily around the circumference of the arterial wall surrounding calcified deposits, while staining was not found in non-calcified sectors. In aortas with more advanced calcification where a calcified plaque occupied the media, the #1 S-Ag procedure stained the calcifying periphery black (* in Fig. 6h-i). Small stones found within the calcified plaques were stained black (arrow in Fig. 6j). The adventitia was not stained.

**Fig. 7:**
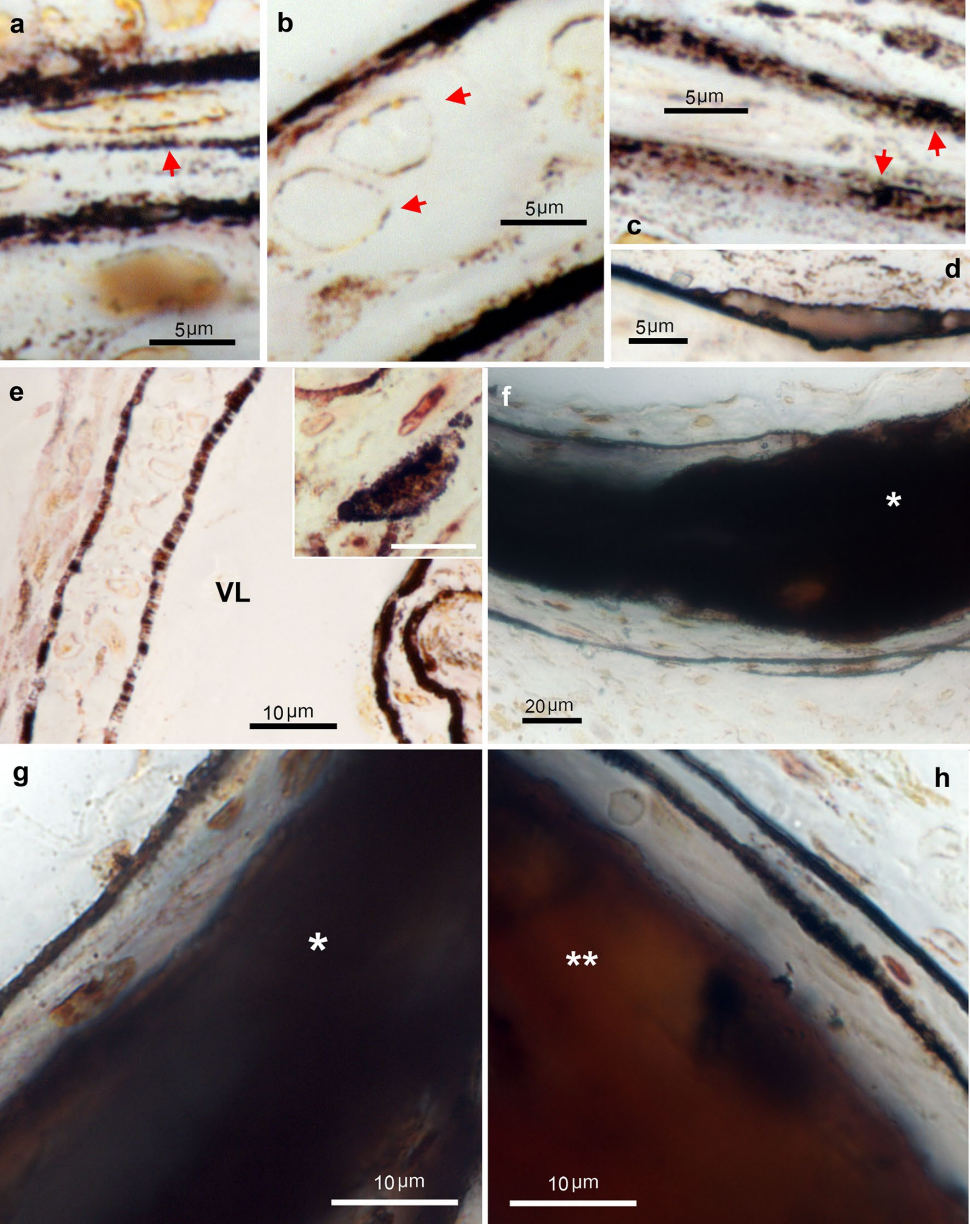
Zn histochemistry of TNAP-OE calcified aortas. #2 EDTA sulfide silver procedure. (**a**) 14 dpn aorta. A VSMC with staining in its outer membrane and a collagen fibril stained with a series of black dots (arrow), both located between two elastic laminae stained black. (**b**) 30 dpn aorta. Two VSMCs (arrows) with linear deposits in its outer membrane located between two elastic laminae stained black. (**c**) 14 dpn aorta. Several elastic laminae with black dots inside (arrows). (**d**) 30 dpn aorta. Elastic lamina stained black with a mineral deposit formed on it which stains light gray. (**e**) 30 dpn aorta. Non-calcified branch of thoracic aorta with initial changes. Its arterial wall contains two elastic laminae that are incompletely stained black. (Inset) A VSMC with linear deposition and surrounding early foci of mineralization. VL indicates vessel lumen. (**f**) 14 dpn aorta. A calcifying area completely stained dark gray. (**g**) 14 dpn aorta. Detail of an elastic lamina stained black and a calcifying area stained gray. (**h**) 30 dpn aorta. Detail of two elastic laminae stained black and a calcified area stained brown with some darker islands. (**a**-**h**) Bright field microscopy

Decalcified aorta sections, #2 E-S-Ag procedure (Fig. 7). As with the #1 S-Ag procedure, the outer membrane of the VSMCs (Fig. 7a-b), elastic laminae (Fig. 7a-h), and some fibrillar collagen (arrow in Fig. 7a) in the non-mineralized matrix of the media were also stained black, indicating that Zn was not extracted at these sites with the applied EDTA treatment. However, small mineral deposits formed on the elastic lamina stained light gray (Fig. 7d). Coincidentally, one of the sections contained a branch of the thoracic aorta in the longitudinal plane. The media of this artery had two elastic laminae and was not calcified but showed early changes. For example, its elastic laminae were partially or completely occupied by a black-stained material (Fig. 7e), and some extracellular foci less than 10 μm in size (inset in Fig. 7e) were found. Calcifying areas in 14 dpn aortas stained fairly uniformly dark gray, but lighter than the black seen in elastic lamina deposits (* in Fig. 7f-g), whereas advanced calcification areas in 30 dpn aortas stained brown (** in Fig. 7h). The distribution of the staining in the artery was more uniform, the patchy part previously seen with the #1 S-Ag procedure disappeared and was almost coincident with the presence of mineral deposits. In both cases, this lighter staining indicated that some Zn was extracted when the mineral was removed with EDTA, i.e., extracted Zn was bound to the mineral (mineral Zn), and that residual Zn (tenacious Zn) was still present in calcification area. The adventitia was not stained, although occasionally some areas of adventitia surrounding a calcified plaque in 30 dpn aortas were stained black.

Taken together, since sulfide exposure and development time were the same for both procedures, the staining of calcified aortas suggested that high concentrations of tenacious Zn (of cellular origin) were present prior to the onset of mineralization, and that there was a higher concentration of mineral Zn than tenacious Zn in the calcified areas.

## Discussion

Zn is a normal trace element found in the five types of vertebrate skeletal and dental tissues: cartilage, bone, cementum, dentin, and enamel. Its concentration has been measured many times using very precise analytical methods in homogenized samples (Gomez et al. 1999). However, what is interesting about the presence of Zn in normal mineralized tissues is not only its average content (≈ 100–300 µg/g), but the fact that Zn is unevenly distributed in these tissues. This was first observed by Haumont (1961) using dithizone staining on epiphyseal cartilage, metaphyseal bone trabeculae and at the calcification front in osteons. Sensitive elemental X-ray microprobe techniques such as (micro)proton induced X-ray emission (µ-PIXE) and synchrotron radiation induced X-ray fluorescence (SR-XRF) have allowed the mapping of Zn, among other elements, in different mineralized tissues with sufficient spatial resolution to confirm this fact. High Zn concentrations are found in the matrix zones just before the onset of mineralization and at the mineralizing sites in epiphyseal cartilage (Doty et al. 1981; Chichocki et al. 1988; Vittur et al. 1992; Silva Barreto et al. 2020) and bone tissues (Doty et al. 1981; Gomez et al. 1999, 2006). In addition, accumulated Zn in the mineralized matrix has been reported as Zn lines and Zn stripes in calcified cartilage (Rizzo et al. 1995; Zöger et al. 2006; Bradley et al. 2007; Kaabar et al. 2010; Raoult et al. 2018; Stock et al. 2022; Kierdorf et al. 2022), bone (Pemmer et al. 2013; Kierdorf et al. 2023), cementum (Martin et al. 2004; Stock et al. 2017; Dean et al. 2018), dentin (Stock et al. 2014; Dean et al. 2023), and enamel (Dean et al. 2019, 2023).

Mineralized tissues are a composite of hydroxyapatite mineral deposited in an organic matrix. Thus, one of the questions to be resolved about the distribution of Zn revealed in these studies is to determine whether Zn is bound to the mineral phase or whether it belongs to an organic molecule involved in the mineralization process, although both pools of Zn can exist simultaneously and in the same location. In fact, at least three Zn metalloenzymes with important roles in the mineralization process have been identified: TNAP, carbonic anhydrase and metalloproteinases (Wuthier 2011).

In the present work, we took advantage of the male TNAP-OE mice, in which TNAP production is very high, to map Zn using the sulfide-silver method in the calcified aorta. Two staining procedures were used to differentiate between mineral and tenacious Zn pools. The reason for using both procedures was to perform a Zn partial extraction by removing mineral Zn with EDTA in mineralized tissues to reveal the tenacious Zn, presumably Zn^2+^ bound to TNAP (Zn-TNAP). Interestingly, in the non-mineralized matrix, both procedures gave the same results, indicating the presence of a pool of tenacious Zn of cellular origin prior to mineralization. The same staining was detected in the outer membrane of VSMCs, in elastic laminae, in some collagen fibers, in tiny foci, and in small stones. These locations suggest that this tenacious Zn belongs to Zn-TNAP, which when produced in large quantities permeates adjacent structures and concentrates in a newly produced calcifiable matrix. However, staining of the area occupied by the mineral deposits was uneven after both procedures. A mineral Zn pool was stained at the calcifying sites with #1 S-Ag procedure. After EDTA treatment, mineral Zn was lost, while the remaining Zn was tenacious Zn. In addition, the more advanced (calcified) the mineral deposit, the less tenacious Zn remained. A plausible interpretation is that the mineral Zn is derived from the Zn lost when TNAP is inactivated, its Zn ions replaced by calcium and transferred to the nascent mineral (Genge et al. 1988), but there was still Zn bound to buried TNAP that has not yet been inactivated.

This interpretation is strengthened by comparing the distribution of tenacious Zn with the histochemical distribution of TNAP, both in the calcified aorta of TNAP-OE mice and in the rat growth plate used as a control. TNAP histochemistry of TNAP-OE has been published previously (Sheen et al. 2015). In this study, their images were obtained from serial cryosections, 10 μm thick, some sections stained for the enzyme TNAP and some sections stained with alizarin red. In the aortas of 14 dpn mice, both stains coincide in the calcifying areas, whereas in those of 30 dpn, the enzyme staining is restricted to the periphery of the already calcified plaques. That is, in one case the TNAP distribution occupies almost the entire thickness of the arterial media, whereas in the other it appears as a ring surrounding the calcified deposits, which are negative. In the rat growth plate, the distribution of histochemical TNAP and tenacious Zn staining also coincided spatially and showed the same sequence. In fact, in the non-mineralized territorial matrix of hypertrophic zone, where TNAP accumulates extracellularly, high concentrations of tenacious Zn were detected. In the calcification nodules found in the calcifying zone, TNAP activity was still present in the initial ones to disappear as mineralization progressed in the confluent calcification nodules, but tenacious Zn still remained.

In addition to Zn ions transferred to the mineral from TNAP, it is possible that some of the mineral Zn at calcifying sites or calcification fronts comes from Zn ions available in the blood. In this context, free Zn ions would behave like other bone-seeking ions since the mineral hydroxyapatite has a high affinity for Zn ions. Indeed, in vitro experiments have demonstrated the uptake of 65Zn by bone powder and inorganic bone (Samachson et al. 1967). Although it is difficult to determine the contribution of one or the other source of Zn ions to mineral Zn, it should be noted that in bone, both mineral Zn and tenacious Zn have been detected at the mineralizing surfaces, but that only mineral Zn is detected at the resting surfaces, as tenacious Zn has already disappeared (Gomez et al. 1999). As a matter of speculation, it is possible to consider that the Zn lines found within the mineralized bone matrix, away from the ion exchange bone surfaces, could correspond to episodes of mineralization restart where high concentrations of TNAP were buried. Thus, TNAP may play a predominant role in *de novo* mineralization, since Zn is more concentrated in the cement lines of the bone remodeling units, in the so-called lines of arrested growth, and in the woven bone, which mineralizes very rapidly (Gomez et al. 1999, 2006; Pemmer et al. 2013; Kierdorf et al. 2023). The absence of active TNAP has the opposite effect. In the model of hypophosphatasia studied by TEM, Anderson et al. (2005) showed in the growth plate that the propagation phase (the proliferation of mineral crystals outside the matrix vesicles) failed, although the matrix vesicles were calcified, and the bone was osteomalacic.

However, the presence of Zn ions in calcification fronts may influence the formation of hydroxyapatite minerals. In vitro studies with isolated matrix vesicles by Wuthier and his group have shown that free Zn^2+^ ions, which are readily extractable and constitute about 40% of the total Zn present in matrix vesicles, play a regulatory role by competing with Ca^2+^ for high-affinity Ca-binding sites on the matrix vesicle membrane or within the matrix vesicle lumen (Sauer et al. 1989; Wuthier 2011). Further in vitro studies by Wuthier have also shown that Zn^2+^ stabilizes a non-crystalline precursor, such as octacalcium phosphate, in matrix vesicles (Sauer et al. 1997; Wuthier 2011). Recently, Zn-hydroxyapatite was characterized by X-ray fluorescence microscopy in early mineral nuclei in osteogenic cell culture experiments (Procopio et al. 2019).

Overall, an interpretation of the results suggests a defined mineralization sequence with the following steps. In male TNAP-OE mice, VSMCs begins to produce and export Zn-TNAP. The initial production of Zn-TNAP is not sufficient for mineralization (aortas of 7 dpn mice still had no calcifications despite early TNAP production (Sheen et al. 2015), as the production of a calcifiable matrix is also required. TNAP is incorporated into the calcifying matrix and acts there. At these sites, TNAP is inactivated and loses its Zn ions, which are transferred to the nascent mineral. Aortic calcification in the TNAP-OE mouse is an extreme pathological model, other medial arterial calcifications progress more slowly and lower levels of TNAP are expected.

A limitation of the current study is that the molecular composition of the calcifying matrix was not determined, although phase contrast imaging and toluidine blue staining suggested concentration of non-collagenous proteins at the calcifying site. In this regard, bone acidic glycoprotein-75 (BAG-75) and bone sialoprotein (BSP) were localized in early mineralization sites as tiny foci and calcospherulites (Gorski et al. 2004; Midura et al. 2007). Analysis of the composition of the calcifying matrix and to which component TNAP may bind requires further investigation.

In conclusion, following the trail of Zn localization, it is suggested that Zn-TNAP, in addition to its catalytic function, has an additional function at calcifying sites as a Ca-binding protein as Zn is progressively substituted by Ca, and that these Zn ions are transferred to the nascent mineral. In a sense, Zn-TNAP is a sacrificial molecule when buried and inactivated in calcifying sites with a functional profile sequestering calcium aiding in the propagation of calcification onto the extracellular matrix.

## Data Availability

No datasets were generated or analysed during the current study.

## References

[CR1] Anderson HC, Harmey D, Camacho NP, Garimella R, Sipe JB, Tague S, Bi X, Johnson K, Terkeltaub R, Millán JL (2005) Sustained osteomalacia of long bones despite major improvement in other hypophosphatasia-related mineral deficits in tissue nonspecific alkaline phosphatase/nucleotide pyrophosphatase phosphodiesterase 1 double-deficient mice. Am J Pathol 166(6):1711–1720. 10.1016/S0002-9440(10)62481-9 15920156 10.1016/S0002-9440(10)62481-9 PMC1602415

[CR2] Arzac A, López-Cepero JM, Babushkina EA, Gomez S (2018) Applying methods of hard tissues preparation for wood anatomy: imaging polished samples embedded in polymethylmethacrylate. Dendrochronologia 51:76–81. 10.1016/j.dendro.2018.08.005 10.1016/j.dendro.2018.08.005

[CR3] Bonucci E, Gomez S (2012) Cartilage calcification. In: Seto J (ed) Advanced topics in Biomineralization. INTECH Open Access, pp 85–110

[CR4] Bradley DA, Moger CJ, Winlove CP (2007) Zn deposition at the bone–cartilage interface in equine articular cartilage. Nucl Instrum Methods Phys Res A 580(1):473–476. 10.1016/j.nima.2007.05.143 10.1016/j.nima.2007.05.143

[CR5] Bromage TG, Gomez S, Boyde A (2018) Imaging hard – inside the skeleton. R Microsc Soc 49:4–31

[CR60] Chichocki T, Gonsior B, Höfert M, Jarczyk L, Raith B, Rokita E, Strazalkowski A, Sych M (1988) Measurements of mineralizationprocess in the femur growth plate and rib cartilage of the mouse using pixe in combination with a proton microprobe. Histochemistry 89:99–104. 10.1007/BF00496591 10.1007/BF00496591 2835343

[CR6] Danscher G, Stoltenberg M, Bruhn M, Søndergaard C, Jensen D (2004) Immersion autometallography: histochemical in situ capturing of zinc ions in catalytic zinc-sulfur nanocrystals. J Histochem Cytochem 52(12):1619–1625. 10.1369/jhc.4A6371.2004 15557216 10.1369/jhc.4A6371.2004

[CR7] De Bernard B, Bianco P, Bonucci E, Costantini M, Lunazzi GC, Martinuzzi P, Modricky L, Panfili E, Pollesello P (1986) Biochemical and immunohistochemical evidence that in cartilage an alkaline phosphatase is a Ca^2+^-binding glycoprotein. J Cell Biol 103(4):1615–1623. 10.1083/jcb.103.4.1615 3771650 10.1083/jcb.103.4.1615 PMC2114361

[CR8] Dean C, Le Cabec A, Spiers K, Zhang Y, Garrevoet J (2018) Incremental distribution of strontium and zinc in great ape and fossil hominin cementum using synchrotron X-ray fluorescence mapping. J R Soc Interface 15(138):20170626. 10.1098/rsif.2017.0626 29321271 10.1098/rsif.2017.0626 PMC5805964

[CR9] Dean MC, Spiers KM, Garrevoet J, Le Cabec A (2019) Synchrotron X-ray fluorescence mapping of Ca, Sr and Zn at the neonatal line in human deciduous teeth reflects changing perinatal physiology. Arch Oral Biol 104:90–102. 10.1016/j.archoralbio.2019.05.024 31176148 10.1016/j.archoralbio.2019.05.024

[CR10] Dean MC, Garrevoet J, Van Malderen SJ, Santos F, Mirazón Lahr M, Foley R, Le Cabec A (2023) The distribution and Biogenic Origins of Zinc in the Mineralised Tooth Tissues of Modern and Fossil hominoids: implications for Life History, Diet and Taphonomy. Biology 12(12):1455. 10.3390/biology12121455 38132281 10.3390/biology12121455 PMC10740576

[CR11] Doty SB, Jones KW, Kraner HW, Shroy RE, Hanson AL (1981) Proton microprobe analysis of zinc in skeletal tissues. Nucl Instrum Methods 181(1–3):159–164. 10.1016/0029-554X(81)90599-1 10.1016/0029-554X(81)90599-1

[CR12] Fedde KN, Blair L, Silverstein J, Coburn SP, Ryan LM, Weinstein RS, Waymire K, Narisawa S, Millán JL, Macgregor GR, Whyte MP (1999) Alkaline phosphatase knock-out mice recapitulate the metabolic and skeletal defects of infantile hypophosphatasia. J Bone Min Res 14(12):2015–2026. 10.1359/jbmr.1999.14.12.2015 10.1359/jbmr.1999.14.12.2015 PMC3049802 10620060

[CR13] Genge BR, Sauer GR, Wu LN, McLean FM, Wuthier RE (1988) Correlation between loss of alkaline phosphatase activity and accumulation of calcium during matrix vesicle-mediated mineralization. J Biol Chem 263(34):18513–18519. 10.1016/S0021-9258(19)81388-1 3192545 10.1016/S0021-9258(19)81388-1

[CR14] Gomez S, Boyde A (1994) Correlated alkaline phosphatase histochemistry and quantitative backscattered electron imaging in the study of rat incisor ameloblasts and enamel mineralization. Microsc Res Tech 29(1):29–36. 10.1002/jemt.1070290105 8000082 10.1002/jemt.1070290105

[CR15] Gomez S, Rizzo R, Pozzi-Mucelli M, Bonucci E, Vittur F (1999) Zinc mapping in bone tissues by histochemistry and synchrotron radiation–induced X-ray emission: correlation with the distribution of alkaline phosphatase. Bone 25(1):33–38. 10.1016/S8756-3282(99)00102-7 10423019 10.1016/S8756-3282(99)00102-7

[CR16] Gomez S, Preoteasa EA, Harangus L, Iordan A, Grambole D, Herrmann F (2006) Micro-PIXE and histochemical studies of Zn and Ca distribution in normal bone. Nucl Instrum Methods Phys Res B 249(1–2):673–676. 10.1016/j.nimb.2006.03.077 10.1016/j.nimb.2006.03.077

[CR17] Gorski JP, Wang A, Lovitch D, Law D, Powell K, Midura RJ (2004) Extracellular bone acidic glycoprotein-75 defines condensed mesenchyme regions to be mineralized and localizes with bone sialoprotein during intramembranous bone formation. J Biol Chem 279(24):25455–25463. 10.1074/jbc.M312408200 15004029 10.1074/jbc.M312408200

[CR18] Haumont S (1961) Distribution of zinc in bone tissue. J Histochem Cytochem 9(2):141–145. 10.1177/9.2.141 13905408 10.1177/9.2.141

[CR19] Hoshi K, Ejiri S, Ozawa H (2001) Localizational alterations of calcium, phosphorus, and calcification-related organics such as proteoglycans and alkaline phosphatase during bone calcification. J Bone Min Res 16(2):289–298. 10.1359/jbmr.2001.16.2.289 10.1359/jbmr.2001.16.2.289 11204429

[CR20] Hoylaerts MF, Van Kerckhoven S, Kiffer-Moreira T, Sheen C, Narisawa S, Millán JL (2015) Functional significance of calcium binding to tissue-nonspecific alkaline phosphatase. PLoS ONE 10(3):e0119874. 10.1371/journal.pone.0119874 25775211 10.1371/journal.pone.0119874 PMC4361680

[CR21] Kaabar W, Gundogdu O, Laklouk A, Bunk O, Pfeiffer F, Farquharson MJ, Bradley DA (2010) µ-PIXE and SAXS studies at the bone–cartilage interface. Appl Radiat Isot 68(4–5):730–734. 10.1016/j.apradiso.2009.09.038 19836249 10.1016/j.apradiso.2009.09.038

[CR22] Kierdorf U, Stock SR, Gomez S, Antipova O, Kierdorf H (2022) Distribution, structure, and mineralization of calcified cartilage remnants in hard antlers. Bone Rep 16:101571. 10.1016/j.bonr.2022.101571 35519288 10.1016/j.bonr.2022.101571 PMC9065892

[CR23] Kierdorf U, Gomez S, Stock SR, Antipova O, Kierdorf H (2023) Bone resorption and formation in the pedicles of European roe deer (Capreolus capreolus) in relation to the antler cycle—A morphological and microanalytical study. J Anat 43:842–859. 10.1111/joa.13908 10.1111/joa.13908 PMC10557394 37278321

[CR24] Martin RR, Naftel SJ, Nelson AJ, Feilen AB, Narvaez A (2004) Synchrotron X-ray fluorescence and trace metals in the cementum rings of human teeth. J Environ Monit 6(10):783–786. 10.1039/B408525F 15480490 10.1039/B408525F

[CR25] Meyer JL (1984) Can biological calcification occur in the presence of pyrophosphate? Arch Biochem Biophys 231(1):1–8. 10.1016/0003-9861(84)90356-4 6326671 10.1016/0003-9861(84)90356-4

[CR26] Midura RJ, Vasanji A, Su X, Wang A, Midura SB, Gorski JP (2007) Calcospherulites isolated from the mineralization front of bone induce the mineralization of type I collagen. Bone 41(6):1005–1016. 10.1016/j.bone.2007.08.036 17936099 10.1016/j.bone.2007.08.036 PMC2238032

[CR27] Millán JL (2006) Mammalian alkaline phosphatases: from biology to applications in medicine and biotechnology. Wiley

[CR28] Millán JL, Whyte MP (2016) Alkaline phosphatase and hypophosphatasia. Calcif Tissue Int 98(4):398–416. 10.1007/s00223-015-0079-1 26590809 10.1007/s00223-015-0079-1 PMC4824800

[CR29] Mornet E, Stura E, Lia-Baldini AS, Stigbrand T, Ménez A, Le Du MH (2001) Structural evidence for a functional role of human tissue nonspecific alkaline phosphatase in bone mineralization. J Biol Chem 276(33):31171–31178. 10.1074/jbc.M102788200 11395499 10.1074/jbc.M102788200

[CR30] Narisawa S, Fröhlander N, Millán JL (1997) Inactivation of two mouse alkaline phosphatase genes and establishment of a model of infantile hypophosphatasia. Dev Dyn 208(3):432–446. 10.1002/(SICI)1097-0177(199703)208:3%3C432::AID-AJA13%3E3.0.CO;2-1 9056646 10.1002/(SICI)1097-0177(199703)208:3<432::AID-AJA13>3.0.CO;2-1

[CR31] Pemmer B, Roschger A, Wastl A, Hofstaetter JG, Wobrauschek P, Simon R, Thaler HW, Roschger P, Klaushofer K, Streli C (2013) Spatial distribution of the trace elements zinc, strontium and lead in human bone tissue. Bone 57(1):184–193. 10.1016/j.bone.2013.07.038 23932972 10.1016/j.bone.2013.07.038 PMC3807669

[CR32] Procopio A, Malucelli E, Pacureanu A, Cappadone C, Farruggia G, Sargenti A, Castiglioni S, Altamura D, Sorrentino A, Giannini C, Pereiro E, Cloetens P, Maier JAM, Iotti S (2019) Chemical Fingerprint of Zn–Hydroxyapatite in the early stages of osteogenic differentiation. ACS Cent Sci 5(8):1449–1460. 10.1021/acscentsci.9b00509 31482128 10.1021/acscentsci.9b00509 PMC6716342

[CR33] Raoult V, Howell N, Zahra D, Peddemors VM, Howard DL, de Jonge MD, Buchan BL, Williamson JE (2018) Localized zinc distribution in shark vertebrae suggests differential deposition during ontogeny and across vertebral structures. PLoS ONE 13(1):e0190927. 10.1371/journal.pone.0190927 29324879 10.1371/journal.pone.0190927 PMC5764331

[CR34] Rizzo R, Grandolfo M, Godeas C, Jones KW, Vittur F (1995) Calcium, sulfur, and zinc distribution in normal and arthritic articular equine cartilage: a synchrotron radiation-induced X‐ray emission (SRIXE) study. J Exp Zool 273(1):82–86. 10.1002/jez.1402730111 7561728 10.1002/jez.1402730111

[CR35] Samachson J, Dennis J, Fowler R, Schmitz A (1967) The reaction of 65Zn with the surfaces of bone and bone mineral. BBA-General Subj 148(3):767–773. 10.1016/0304-4165(67)90050-5 10.1016/0304-4165(67)90050-5

[CR36] Sauer GR, Adkisson HD, Genge BR, Wuthier RE (1989) Regulatory effect of endogenous zinc and inhibitory action of toxic metal ions on calcium accumulation by matrix vesicles in vitro. Bone Min 7(3):233–244. 10.1016/0169-6009(89)90080-9 10.1016/0169-6009(89)90080-9 2611445

[CR37] Sauer GR, Wu LN, Iijima M, Wuthier RE (1997) The influence of trace elements on calcium phosphate formation by matrix vesicles. J Biol Chem 65(1):57–65. 10.1016/S0162-0134(96)00080-3 10.1016/S0162-0134(96)00080-3 8987171

[CR38] Schenk R, Olah A, Herrmann W (1984) Preparation of calcified tissues for light microscopy. In: Dickson G (ed) Methods of calcified tissue Preparation. Elsevier, pp 1–56

[CR39] Sheen CR, Kuss P, Narisawa S, Yadav MC, Nigro J, Wang W, Chhea N, Sergienko EA, Kapoor K, Jackson MR, Hoylaerts MF, Pinkerton AB, O’Neill WC, Millán JL (2015) Pathophysiological role of vascular smooth muscle alkaline phosphatase in medial artery calcification. J Bone Min Res 30(5):824–836. 10.1002/jbmr.2420 10.1002/jbmr.2420 PMC4406354 25428889

[CR40] Silva Barreto I, Le Cann S, Ahmed S, Sotiriou V, Turunen MJ, Johansson U, Rodriguez-Fernandez A, Grünewald TA, Liebi M, Nowlan NC, Isaksson H (2020) Multiscale characterization of embryonic long bone mineralization in mice. Adv Sci 7(21):2002524. 10.1002/advs.202002524 10.1002/advs.202002524 PMC7610310 33173750

[CR41] Stagni N, Furlan G, Vittur F, Zanetti M, De Bernard B (1979) Enzymatic properties of the Ca^2+^-binding glycoprotein isolated from preosseous cartilage. Calcif Tissue Int 29(1):27–32. 10.1007/BF02408052 116741 10.1007/BF02408052

[CR42] Stock SR, Deymier-Black AC, Veis A, Telser A, Lux E, Cai Z (2014) Bovine and equine peritubular and intertubular dentin. Acta Biomater 10(9):3969–3977. 10.1016/j.actbio.2014.05.027 24911530 10.1016/j.actbio.2014.05.027 PMC4123743

[CR43] Stock SR, Finney LA, Telser A, Maxey E, Vogt S, Okasinski JS (2017) Cementum structure in Beluga whale teeth. Acta Biomater 48:289–299. 10.1016/j.actbio.2016.11.015 27836805 10.1016/j.actbio.2016.11.015

[CR44] Stock SR, Morse PE, Stock MK, James KC, Natanson LJ, Chen H, Shevchenko PD, Maxey ER, Antipova OA, Park JS (2022) Microstructure and energy dispersive diffraction reconstruction of 3D patterns of crystallographic texture in a shark centrum. J Med Imag Radiat Oncol J Med Imaging 9(3):031504. 10.1117/1.JMI.9.3.031504 10.1117/1.JMI.9.3.031504 PMC8809398 35127969

[CR45] Väänänen HK (1980) Immunohistochemical localization of alkaline phosphatase in the chicken epiphyseal growth cartilage. Histochemistry 65(2):143–148. 10.1007/BF00493162 6987196 10.1007/BF00493162

[CR46] Vittur F, De Bernard B (1973) Alkaline phosphatase activity associated to a calcium binding glycoprotein from calf scapula cartilage. FEBS lett 38(1):87–90. 10.1016/0014-5793(73)80520-4 4204053 10.1016/0014-5793(73)80520-4

[CR47] Vittur F, Tuniz C, Paoletti S, Rizzo R, Jones KW (1992) Elemental analysis of growth plate cartilage by synchrotron-radiation-induced X-ray emission (SRIXE). Biochem Biophys Res Commun 188(3):1010–1017. 10.1016/0006-291X(92)91332-K 1445337 10.1016/0006-291X(92)91332-K

[CR48] Wuthier RE (2011) Matrix vesicles: structure, composition, formation and function in calcification. Front Biosci 16:2812–2902. 10.2741/3887 10.2741/3887 21622210

[CR49] Zöger N, Roschger P, Hofstaetter JG, Jokubonis C, Pepponi G, Falkenberg G, Fratzl P, Berzlanovich A, Osterode W, Streli C, Wobrauschek P (2006) Lead accumulation in tidemark of articular cartilage. Osteoarthritis Cartilage 14(9):906–913. 10.1016/j.joca.2006.03.001 16678451 10.1016/j.joca.2006.03.001

