# Correction: Zinc-alkaline phosphatase at sites of aortic calcification

**DOI:** 10.1007/s10735-024-10265-7

**Published:** 2024-09-14

**Authors:** Santiago Gomez, José Luis Millán

**Affiliations:** 1 Departamento Anatomía Patológica, Facultad de Medicina, Universidad de Cádiz, Plaza Fragela 9, Cádiz, 11003 Spain; 2 Sanford Children’s Health Research Center, Sanford Burnham Prebys Medical Discovery Institute, La Jolla, CA USA

**Correction to: Journal of Molecular Histology**

10.1007/s10735-024-10207-3

In this article, there was a typo in the references ‘Bromage TG, Gomez S, Boyde A Imaging hard–inside the skeleton. InFocus (Proceedings of the royal microscopical Society) 49:4–31 and Chichocki T, Gonsior B, Höfert M, Jarczyk L, Raith B, Rokita E, Strazalkowski A, Sych M (2018) (1988) Measurements of mineralizationprocess in the femur growth plate and rib cartilage of the mouse using pixe in combination with a proton microprobe. Histochemistry 89:99–104. 10.1007/BF00496591. The corrected references should read as below:

Bromage TG, Gomez S, Boyde A (2018) Imaging hard–inside the skeleton. InFocus (Proceedings of the royal microscopical Society) 49:4–31.

Chichocki T, Gonsior B, Höfert M, Jarczyk L, Raith B, Rokita E, Strazalkowski A, Sych M (1988) Measurements of mineralizationprocess in the femur growth plate and rib cartilage of the mouse using pixe in combination with a proton microprobe. Histochemistry 89:99–104. 10.1007/BF00496591.

The original article has been corrected.

